# Supplementary material for: Are carbon emissions trading and green financial instruments synergistic? -Comprehensive quantitative research based on content analysis
Source: PLoS One. 2024 Mar 7;19(3):e0298601. doi: 10.1371/journal.pone.0298601 (PMC10919601; doi:10.1371/journal.pone.0298601)
Supplement: S1 Data — (ZIP) [file pone.0298601.s001.zip › Data/地级市Z_F工作报告.zip!/║╙─╧/Σ≡║╙╩╨/└┤╫╘╣1⁄2╓┌║┼í╛┬φ┐╦╩2╛▌═°í┐_2013.docx]

各位代表:

现在，我代表市人民政府，向大会作工作报告，请予审议，并请各位政协委员和其他列席人士提出意见。

一、2012年工作回顾

--全市经济较快增长。全市生产总值812.4亿元，同比增长12.1%，增速居全省第3位;地方公共财政预算收入41.6亿元，比上年净增7.9亿元，增长23.5%，增速居全省第5位;税收收入占地方公共财政预算收入的比重达到80.2%，居全省第2位;固定资产投资526.8亿元，增长23.7%，增速居全省第7位;社会消费品零售总额294.4亿元，增长15.9%，增速居全省第5位;规模以上工业利润228.3亿元，增长21.6%，增速居全省第4位。经济运行质量持续提升，主要经济指标增速进入全省第一方阵。

--发展动力更加强劲。各项改革深入推进，30家企业实施了战略重组;双汇集团整体上市，成为全省市值最大的上市公司;漯河银行挂牌运营，市农村发展投资有限公司正式成立，农村信用社达标升级全面完成，城投12亿元二期债券成功发行;城市公交改革实现新突破，市公交集团成功组建。开放招商成果丰硕，全年实际利用外资6.2亿美元、实际利用省外资金150亿元、外贸出口总额2.5亿美元;与13个省直部门签订了战略合作协议，争取上级政策性资金75.6亿元;成功举办了第十届中国(漯河)食品博览会，中国食品名城的知名度和影响力进一步扩大。重点项目建设力度加大，52个项目竣工投运，64个项目开工建设，140个市定重点项目完成投资208亿元，省定重点项目投资完成率居全省第1位。产业集聚区建设加快推进，6个产业集聚区全部实现扩区，新增发展面积45.7平方公里，完成投资380亿元，实现主营业务收入1200亿元，从业人员达到12.6万人。全年财政总收入83.5亿元，财政总支出达到111.2亿元，财政保障能力显著增强。

--"三农"工作全面加强。全市发放各类涉农资金24.3亿元，广大农民得到更多实惠。全年建成高标准粮田33.5万亩，粮食总产173万吨、再创历史新高，蔬菜种植面积达90万亩，完成植树造林面积4万亩，农村土地流转规模达到84.7万亩，新增国家级标准化规模养殖场5个、省级农业产业化重点龙头企业9家、农民专业合作社200个，农业产业化经营水平进一步提升，我市成为"国家级绿色食品原料(辣椒)标准化生产基地"创建城市、"全省畜产品质量安全监管体系建设试点市"。农村生产生活条件明显改善，新发展有效灌溉面积10万亩、旱涝保收田9万亩，新建农村户用沼气池8025座，新建、改造农村公路140公里，新解决18.3万人饮水安全问题，新改造农村危房7000户。实现1.8万农村贫困人口稳定脱贫。补贴大型农机具3325台(套)，农机化装备水平明显提高，我市成为"创建全国农业机械化示范市"。秸秆禁烧及综合利用工作成效显著，主要农作物机收率、还田率、秸秆利用率均创历史最好水平。新开工建设新型农村社区33个，建成及在建住房2.3万套，5600多户入住新居，新型农村社区建设实现新突破。

--城市形象大幅提升。沙澧河开发建设一期工程全面完成，形成了长达36公里的生态休闲沿河景观带;沙澧河风景区成功创建国家4A级景区，被确定为全省第一个国家体育公园，已成为漯河的一张靓丽名片。高铁客运站通车运营，交通路、建设路改造提前完成，白云山路升级改造、交通路沙河桥加宽加固等工程推进顺利。旧城区和城中村改造加快推进，改造背街小巷26条。供气、供排水等基础设施建设力度加大，城市功能得到完善。西城区路网框架基本形成，漯河新区各项前期工作全面铺开，商务中心区和特色商业区规划建设走在全省前列。城市管理综合提升行动深入开展，社会关注的交通拥堵、城市内涝等问题得到有效缓解，数字化城市管理平台建成投用，国家卫生城市创建工作强力推进，我市荣获首批"河南省最佳宜居城市"称号。

--民生质量显著提高。全市各级财政用于民生的支出达到86亿元，占公共财政预算支出的77.3%。省定十项民生工程、市定15件民生实事全面完成。城镇居民人均可支配收入19136元、农民人均纯收入8755 元，分别增长12.6%和13.7%，城乡居民收入实现了与经济同步增长。城乡居民存款余额达到367.4亿元，增长23.3%，增速居全省第1位。全年城镇新增就业7.4万人、再就业2.6万人，新增农村劳动力转移就业3.9万人。发放个人住房公积金贷款5.4亿元，建设各类保障性住房1.2万套。城乡居民社会养老保险实现全覆盖，企业职工工资正常增长机制全面建立，机关公务员津补贴和事业单位绩效工资改革政策全面落实，城镇居民医疗保险大病补偿、新农合大病补偿、城乡低保标准、农村五保供养标准和企业退休人员养老金全面提高，社会保障能力全面提升。

--社会事业持续进步。质量立市战略深入实施，科技、人才工作得到加强，新建院士工作站3个，新认定国家级高新技术企业2家，我市成为首批"全国质量强市示范城市"创建城市、首批"国家智慧城市"试点城市。教育事业全面发展，全市新建幼儿园75所、改扩建43所，市区新建中小学4所，普通高考本科上线人数继续保持在万人以上，职业教育攻坚计划全面完成。城乡医疗卫生服务体系不断完善，县级公立医院综合改革稳步推进，1316个村卫生室全部纳入基本药物制度实施范围。我市连续6次荣获"全国无偿献血先进市"称号。文化旅游业加快发展，农家书屋实现了全覆盖，"幸福漯河健康舞"成为新的群众文化品牌，小商桥景区、周彦生艺术研究院、金凤凰文化产业园建成开放。成功举办世界女排大奖赛等9项省级以上体育赛事，全民健身运动广泛开展。计划生育、环境保护和土地管理三大基本国策得到较好落实。统计、审计、人防、气象等工作进一步加强，广播影视、新闻出版、外事侨务、民族宗教、史志档案、慈善事业等取得新成效，妇女、未成年人、老年人和残疾人合法权益得到保障。驻漯解放军、武警官兵和民兵预备役人员在支持地方建设中作出了重要贡献，军民融合式发展进一步深化，我市荣获"全国双拥模范城"称号。

民主法制建设不断加强，自觉接受人大和政协监督，672件人大代表建议和政协提案全部办结;依法治市和法治政府建设深入推进，我市被评为"全国法治城市创建工作先进城市"。廉政建设和反腐败斗争力度加大。精神文明建设成效显著。信访形势、安全生产形势持续平稳，食品药品监管水平进一步提高，平安漯河建设不断深化，社会管理综合治理和应急管理得到加强，公众安全感指数居全省前列，全市社会大局持续和谐稳定。

各位代表!过去的一年，是新一届政府的开局之年。一年来，我们肩负着加快漯河发展的重任，承载着全市人民的热切期盼，始终不敢有丝毫懈怠，着力突出重点、破解难点、打造亮点，向全市人民交了一份来之不易的答卷。

面对日益激烈的区域竞争，我们坚持高端定位、加压奋进。明确提出了经济发展速度、改革开放水平、"三化"协调发展、群众幸福指数"四个全省领先"奋斗目标，各县区明争暗赛、竞相发展，各部门立足大局、强化服务，以更加强烈的紧迫感、使命感和责任感，奋力拼搏，争先进位，致力在新一轮竞争中勇立潮头、在加快"三化"进程中争当先锋、在中原经济区建设中实现跨越。

面对严峻的宏观经济形势，我们坚持科学谋划、积极应对。多次召开市政府常务会、经济运行会、专题推进会，加强对经济形势的分析预判，及时出台保持经济平稳较快增长的15条意见，成立了全市经济运行协调督导领导小组，深化企业服务活动，有效遏制了经济下滑态势，全市经济保持了平稳较快增长。

面对繁重的发展任务，我们坚持团结协作、狠抓落实。在招商引资、项目建设等重点工作中，在温家宝总理视察漯河、第十届中国(漯河)食品博览会等重大活动中，各级各部门密切配合、协同作战，特别是通过适时组织重点项目和产业集聚区观摩、新型农村社区观摩、重点项目集中开工、大招商"季度赛"等一系列活动，全市上下进一步增强了压力、增添了动力、激发了活力，形成了心齐、风正、劲足的良好局面。

面对各种热点难点问题，我们坚持改革创新、勇于担当。着力破解影响发展的土地、资金、用工等瓶颈制约，全面启动并强力推进社会各界极为关注的城市公交改革、城市总规修编和城市各专项规划的编制，大力推进城市管理体制改革，简政放权，更好地调动了各区发展经济和管理城市的积极性，持续深化户籍管理制度改革，进一步规范财政资金管理，扎实开展"双整双促双承诺"、涉企科室评议等优化发展环境活动，实现了体制机制新突破，累积了加快发展的正能量。

各位代表!过去的一年，我们在困难中实现了较快发展，在奋进中坚定了必胜信心，在实干中铸就了新的辉煌。这是省委、省政府和市委正确领导的结果，是市人大、市政协监督支持的结果，是全市人民团结奋斗、顽强拼搏的结果。在此，我代表市人民政府，向工作在各条战线上的工人、农民、知识分子、干部，向驻漯解放军、武警官兵、公安民警以及各界人士，致以崇高的敬意!向所有关心支持漯河发展的同志们、朋友们，表示诚挚的感谢!

各位代表!在看到成绩的同时，我们也清醒地认识到，经济社会发展中还存在不少问题和不足，政府工作与人民的期待还有一定差距。主要表现在:产业层次总体上较为低端，创新能力不强，战略性新兴产业规模较小，服务业占比不高;城镇化水平低，城镇功能还不完善;资源环境约束加剧，经济发展的瓶颈依然存在;城市供热、物业管理等涉及群众切身利益的问题尚未得到很好解决，改善民生和加强社会管理任务依然繁重;政府职能转变还有差距，一些部门官僚主义、形式主义问题突出，一些干部创新意识不强、进取精神不足，推诿扯皮、效率低下，因循守旧、不敢担当，极少数干部奢侈浪费、消极腐败现象依然存在。对此，我们必须始终保持清醒忧患，决不回避、决不推卸、决不懈怠，努力把各类问题解决好、把各种矛盾化解好、把漯河的事情办得更好!

二、2013年工作安排

今年是全面贯彻落实党的十八大精神和实施《中原经济区规划》的开局之年，是实施"十二五"规划承前启后的关键一年，也是为全面建成小康社会奠定坚实基础的重要一年，做好今年的工作意义重大。

今年政府工作的总体要求是:坚持以科学发展为主题，以加快转变经济发展方式为主线，以提高质量和效益为中心，以稳中求进、稳中求好、稳中求快为主基调，深入实施开放招商、改革创新、项目建设、质量立市、创优环境"五大带动"，着力调整优化经济结构，着力加快"三化"协调发展，着力保障和改善民生，努力保持经济又好又快发展和社会和谐稳定。

今年经济社会发展的主要预期目标是:生产总值增长11%;地方公共财政预算收入增长12%;全社会固定资产投资增长22%;社会消费品零售总额增长16%;外贸出口总额增长15%以上;实际利用外资增长15%以上;城镇居民人均可支配收入、农民人均纯收入实际增长10%以上，力争与经济增长同步;城镇新增就业4万人;居民消费价格涨幅、人口自然增长率控制在省定目标以内;省定节能减排目标全面完成。

围绕上述目标，今年要着重抓好六项工作。

(一)坚定不移加快开放招商和项目建设，着力增创经济社会发展新优势。坚持以开放招商为统领，锲而不舍强力招商，持之以恒狠抓项目。今年，实际利用外资7亿美元，实际利用省外资金175亿元，完成外贸出口总额3亿美元;安排市级重点项目150个，完成投资220亿元，带动全市固定资产投资完成600亿元以上。

拓宽开放领域。持续扩大工业开放，加快推进服务业、现代农业、社会事业和基础设施等领域的开放;毫不动摇鼓励、支持、引导非公有制经济发展，大力引进外来资本和民间资本，着力构建全方位、宽领域、多层次对外开放新格局。

提高招商水平。强化产业招商，重点围绕食品、盐化工、电子信息等产业，制定专项行动方案，搞好对外推介，实现集群式招商。突出以商招商，充分发挥现有企业的招商主体作用，与境内外大企业、大财团进行战略合作，实现增资扩产。实施园区共建，推动各县区与沿海地区的政府、商会、行业协会和实力雄厚的投资者，开展战略合作，推进园区整体开发。积极组织参加各种对外招商活动，精心举办好第十一届中国(漯河)食品博览会。完善招商引资考评办法，充分调动各方面的积极性，在全市上下持续掀起开放招商新热潮。

狠抓项目建设。强化项目支撑、投资拉动，高起点、高标准推进项目建设。新开工建设富铭新材料、旺旺工业园、逍白线改造等一批重点项目，加快推进双汇第二工业园二期、大健康工业园、统一工业园、沙颍河复航工程等一批在建重点项目，建成可口可乐二期、嘉吉果糖、氯化法钛白粉等一批重点项目，完成107国道改造和时南线改造主体工程。瞄准产业转移趋势和国家产业政策取向，谋划储备一批投资大、质量高、带动力强的重大项目。抢抓国家、省里扩大投资的机遇，快速行动，加强对接，争取上级更多的项目。完善项目推进机制，加大协调服务力度，着力破解瓶颈制约，全力以赴抓前期、强督导、快推进，以项目的大建设带动大投资、推动大发展。

(二)坚定不移加快新型城镇化建设，着力构建城乡一体化发展新格局。坚持以新型城镇化为引领，推进产业集聚、人口集中、土地集约，加快城镇化进程，提升城镇化质量。

进一步完善城乡规划体系。编制完成城市总体规划和28项城市专项规划，开展市区重点区域控制性详细规划编制工作，完善村镇体系规划，加快编制新型农村社区规划，形成覆盖全市、层次分明、相互协调的城乡规划体系。

加大中心城市建设力度。稳妥有序推进沙澧河二期开发建设，开工建设"引澧入沙"综合开发工程。抓好市区路桥建设，新建、改造城市道路32条，完成交通路沙河桥加宽加固工程，开工建设解放路沙河桥、牡丹江路沙河桥、太白山路沙河桥和澧河桥、中山路上跨立交桥、人民路火车站区域和新天地区域的地下通道。强力推进旧城区和城中村改造，加快老城区企业"退二进三"步伐，抓好老旧市场升级改造和老旧小区整治。加强城市基础设施建设，在市区布局建设4个热源点，新铺设供热管网30公里;新铺设燃气管网22公里;改扩建第三水厂，建设第五水厂;新建星级公厕40座，全面消除市区旱厕;新建、改造垃圾中转站20座。深化城市公交改革，优化公交线路，加快公交场站建设。大力度推进西城区建设，全年完成投资30亿元以上，重点抓好道路、商务楼、社区和城市规划馆等项目建设。紧紧围绕把漯河新区建成"城乡一体化先行区、现代化复合型功能区、对外开放示范区、中国食品名城核心区、豫中南综合交通枢纽和区域商贸物流中心"这一战略定位，举全市之力，加快推进新区开发建设。今年，要完成新区发展战略策划和各项规划编制，建立管理体制机制，搭建投融资平台，挂牌运营农发行新区支行，开工建设牡丹江路等基础设施，启动双汇总部港等重点项目，确保新区开发建设开好局、起好步。深入开展国家卫生城市创建工作，着力实施卫生清洁、交通秩序整治等七大专项行动，力争年内通过国家卫生城市初检验收。

抓好县城和小城镇建设。进一步明确临颍、舞阳两组团的产业定位、功能分区，加快产业和人口向组团集聚。强化组团的基础设施和公共服务体系建设，促进中心城市组团式联动发展。积极推进重点乡镇内涵式发展，不断提升城镇的综合承载力和产业支撑力。加大撤乡建镇力度，力争两年内实现大的突破。

扎实推进新型农村社区建设。不断完善政策体系，建立多元投入机制，探索多种建设模式，发挥典型带动作用，全面提升建设档次和水平。加大产业培育力度，加快促进人口集聚。统筹处理好"建新"和"拆旧"的关系，及时腾出和复垦土地。今年，新开工建设吸纳超5000人的新型农村社区10个以上，建成及在建住房达到3万套以上。

加强生态文明建设。更加注重节约资源和保护环境，着力推进绿色发展、循环发展、低碳发展，努力建设美丽漯河。落实节能减排措施，抓好重点减排工程，完成市污泥处置厂建设，各产业集聚区的污水处理厂全部建成投运。加强农村环境连片综合整治，控制面源污染。加快"中原经济区城乡绿化一体化示范市"建设，加强道路林网、农田林网建设，开工建设沙河湿地公园，规划建设郝家台遗址公园。通过全市人民的共同努力，把漯河建成天更蓝、地更绿、水更净、空气更清新的美丽家园。

(三)坚定不移加快工业强市建设，着力实现产业转型升级新突破。坚持以新型工业化为主导，抓服务、破瓶颈，抓投入、强后劲，抓重组、增活力，全面提升工业整体竞争力。

加快产业集聚区发展。围绕主导产业，出台扶持政策，加大项目入驻力度，促进产业集群发展，切实增强带动能力。抓好产业集聚区扩区后的规划修编工作，加强基础设施建设，高标准规划建设集商住、服务、生活于一体的服务区，抓好投融资、信息化、土地储备、劳动保障、公共创新等平台建设。全年6个产业集聚区完成固定资产投资300亿元以上，规模以上企业主营业务收入达到1500亿元。

做大做强主导产业。大力推进食品产业改造提升，依托中国百强食品工业园招引一批食品名企入驻，培育食品机械制造等配套产业，形成食品产业突出、名品名企集聚、相关产业配套的发展格局。加快盐化工产业链向高端延伸，打造全省重要的盐化工基地。支持造纸业发展，进一步优化产品结构，巩固其行业优势地位。大力发展电子信息产业，加快建设智能终端产业示范园，重点打造东兴电子工业园和临颍电子电器产业基地，力争电子信息产业快速形成规模。

壮大骨干企业群体。实施"百户重点企业培育行动计划"，全力支持双汇、烟厂等支柱企业和旺旺、曙光医疗等高成长性企业快速发展，全年百户重点企业销售收入突破1500亿元。实施"百家小微企业提升行动计划"，在全市筛选100家成长潜力大、发展势头好的小微企业，倾斜政策，加强扶持，加快培育成我市工业发展的第二、第三梯队。

强化技术创新和企业服务。突出企业创新主体地位，支持企业建设创新平台。制定"科技创新三年培育计划"，培育一批省级创新型企业。立足主导产业，组建产业技术创新联盟，推动企业技术进步和产业结构优化升级。继续实施市级领导分包重点企业、百名优秀干部入企帮扶制度，建立长效服务机制。积极推进中小微企业的金融服务体系建设，有效破解融资难题。引进国内知名专业团队，强化企业上市辅导培育，努力实现企业上市新突破。
